# Perspective on Corticosteroid Use and Interleukin-1 Inhibition With Anakinra in Pericarditis During Pregnancy

**DOI:** 10.1016/j.jacadv.2026.102655

**Published:** 2026-03-19

**Authors:** Lucia Trotta, Ruggiero Mascolo, Savino Paoletta, Antonio Brucato

**Affiliations:** a Internal Medicine, Fatebenefratelli Hospital, University of Milan, Milan, Italy; b Division of Internal Medicine, ASST Fatebenefratelli Sacco, Department of Biomedical and Clinical Sciences, University of Milan, Milan, Italy

We read with interest the report by Schmitz et al^1^ describing management of acute pericarditis during pregnancy. The authors address a challenging and understudied setting, providing valuable insights.

In their cohort, corticosteroids were frequently used, with a median starting dose of 20 mg, reflecting current practice in pericarditis. However, this dosage is now considered too high in pregnancy. The 2020 American College of Rheumatology Guideline for the Management of Pregnancy in Rheumatic Diseases recommends continuing low-dose glucocorticoid treatment (≤10 mg daily of prednisone) during pregnancy and strongly recommends tapering higher doses of <20 mg daily of prednisone, adding pregnancy-compatible glucocorticoid-sparing agents if necessary.^2^ Similarly, the British Society Guidelines recommend that the dose of prednisolone should be <20 mg/day and tapered to the minimum effective dose, in conjunction with steroid-sparing drugs compatible with pregnancy.^3^ In fact, prolonged exposure to systemic corticosteroids is associated with well-recognized maternal and fetal risks, including gestational diabetes, hypertension, intrauterine growth restriction, and preterm delivery.

In this context, emerging evidence supports the role of interleukin-1 blockade with anakinra as a potential steroid-sparing strategy in selected patients with pericarditis during pregnancy.^4^ Notably, anakinra is a recombinant analog of the endogenous interleukin-1 receptor antagonist, which is physiologically present in human circulation and detectable in maternal milk.^5^ We reported favorable maternal and neonatal outcomes in 17 pregnancies in 14 women with recurrent pericarditis treated with anakinra throughout pregnancy and breastfeeding in a recent multinational case series.^4^ No congenital anomalies, neonatal infections, or drug-related adverse events were observed. Disease recurrences were effectively managed through anakinra dose adjustments, achieving sustained inflammatory control and minimizing prolonged corticosteroid exposure.^3^

Randomized trials are unlikely in this setting, and experiences such as those reported by Schmitz are very useful. Accumulating real-world evidence underscore 2 issues: 1) the dose of prednisone should be <20 mg/day and tapered to the minimum effective dose to control maternal disease during pregnancy, in conjunction with steroid sparing drugs compatible with pregnancy; and 2) in this context, anakinra may be very useful to reduce the dose of corticosteroids in women with recurrent pericarditis planning a pregnancy when previous therapies failed.
